# Monitoring of ultra- and diafiltration processes by Kalman-filtered Raman measurements

**DOI:** 10.1007/s00216-022-04477-7

**Published:** 2023-01-18

**Authors:** Laura Rolinger, Jürgen Hubbuch, Matthias Rüdt

**Affiliations:** 1 Institute of Engineering in Life Sciences, Section IV: Biomolecular Separation Engineering, Karlsruhe Institute of Technology (KIT), Karlsruhe, Germany; 2 Hoffmann-La Roche AG, Basel, Switzerland; 3 Haute Ecole d’Ingénierie (HEI), HES-SO Valais-Wallis, Rue de l’industrie 19, Sion, Switzerland

**Keywords:** UF/DF, PAT, Raman spectroscopy, EKF

## Abstract

Monitoring the protein concentration and buffer composition during the Ultrafiltration/Diafiltration (UF/DF) step enables the further automation of biopharmaceutical production and supports Real-time Release Testing (RTRT). Previously, in-line Ultraviolet (UV) and Infrared (IR) measurements have been used to successfully monitor the protein concentration over a large range. The progress of the diafiltration step has been monitored with density measurements and Infrared Spectroscopy (IR). Raman spectroscopy is capable of measuring both the protein and excipient concentration while being more robust and suitable for production measurements in comparison to Infrared Spectroscopy (IR). Regardless of the spectroscopic sensor used, the low concentration of excipients poses a challenge for the sensors. By combining sensor measurements with a semi-mechanistic model through an Extended Kalman Filter (EKF), the sensitivity to determine the progress of the diafiltration can be improved. In this study, Raman measurements are combined with an EKF for three case studies. The advantages of Kalman-filtered Raman measurements for excipient monitoring are shown in comparison to density measurements. Furthermore, Raman measurements showed a higher measurement speed in comparison to Variable Pathlength (VP) UV measurement at the trade-off of a slightly worse prediction accuracy for the protein concentration. However, the Raman-based protein concentration measurements relied mostly on an increase in the background signal during the process and not on proteinaceous features, which could pose a challenge due to the potential influence of batch variability on the background signal. Overall, the combination of Raman spectroscopy and EKF is a promising tool for monitoring the UF/DF step and enables process automation by using adaptive process control.

**Supplementary Information:**

The online version contains supplementary material available at 10.1007/s00216-022-04477-7.

## Introduction

Biopharmaceuticals are an important asset to the modern pharmaceutical industry due to their potential to address diseases that were previously difficult to treat and, from an economical point of view, due to their high retail prices [1, 2]. Biopharmaceuticals are most often produced by genetically modified cells in bioreactors [3]. After the cultivation, the biopharmaceuticals are purified during the Downstream Processing (DSP) to a target purity to allow an administration to patients. The DSP most importantly incorporates centrifugation, chromatography, and filtration steps [4].

Among the listed DSP unit operations, Cross-flow Filtration (CFF) is used at least once at the end of the production process to set the final protein concentration and transfer biopharmaceuticals into their formulation buffers [5]. The unit operation uses the large hydrodynamic diameter of proteins to retain them in a recycling system, while buffer components, water, and contaminants are forced through a membrane [6]. Typically, the process is performed in multiple steps. First, the biopharmaceutical is concentrated to an intermediate concentration to reduce the initial volume during a first Ultrafiltration (UF) step. Second, a buffer exchange into the formulation buffer is performed during a Diafiltration (DF) step. Normally, the protein concentration remains stable during this step. A preset volume of formulation buffer (e.g., five times the pool volume) is forced over the membrane to ensure a sufficient depletion of the original buffer. In the case of highly concentrated drug substance solutions, a second UF step is subsequently used to concentrate the biopharmaceutical to its target concentration. The second UF step helps to avoid concentration-related gel formation on the membrane during the previous DF step which would decrease the process performance [5]. Additionally, the DF buffer should be designed to reduce the viscosity of the protein solution to reduce the process time of the second UF step [7].

It is common practice during process development and production to rely on mass balances to monitor the progress of the Ultrafiltration/Diafiltration (UF/DF) steps. For example, the DF step is completed if a certain number of DF volumes have been exchanged. Typically, either scales or mass flow meters (e.g., Coriolis sensors) are used as input for the mass balances. While this allows monitoring the overall progress, it is only an indirect measurement of important metrics such as the exchange of buffering species or the current protein concentration. During development, effects such as the Donnan effect [8] and protein adsorption to the CFF membrane [9] need to be investigated. The Donnan effect may prevent the full depletion of product counter ions due to the build-up of an electrostatic potential over the membrane [10]. Some buffer components thus might be inadvertently retained despite a diafiltration step. Protein adsorption on membranes is caused by concentration polarization [9]. Proteins are advectively transported to the membrane reaching very high concentrations. Consequently, the proteins may adsorb or interact with other proteins. The conditions may lead to protein aggregation, decreased permeate flow and protein loss.

Off-line analytics are often required to measure the concentration of the target protein and buffer components. In-line and real-time measurements promise to more easily detect said effects and may potentially speed up process development [11]. During production, a control strategy needs to ensure that the product concentration is within the normal operating range during DF and that the final protein concentration complies with the specifications. Especially for subcutaneously administered monoclonal Antibodys (mAbs), the high concentrations and low volumes make an in-line control attractive. Not achieving the required protein concentrations during DF and at the end of the process may result in reprocessing or even batch loss. In-line and real-time measurements can reduce this risk and are useful to reduce manual interventions. Additionally, real-time measurements can be used to automate the process resulting in better-controlled processes and improved process times.

Previously, several studies have already investigated Process Analytical Technology (PAT) methods for the UF/DF step. Most studies focused on monitoring at least one of the typical critical quality attributes (protein concentration, excipient concentration, and aggregate content) during UF/DF. Rolinger et al. used a combination of multiple process analyzers, which were mathematically connected, to calculate protein concentration, buffer exchange progress, and the apparent molecular weight [12]. While in this approach a density signal allowed to monitor the buffer exchange, the effect of the changing protein concentration was neglected, thus potentially resulting in a biased observation. Furthermore, the apparent molecular weight is based on light-scattering measurements which does not allow the independent quantification of aggregates and monomeric species. West et al. [13] used on-line Ultra High Performance Liquid Chromatography (UHPLC) to monitor the protein concentration, aggregate content, and the UV-active excipients. The benefit of an on-line UHPLC is the measurement accuracy, the downsides are long measurement times (5min to 15min), the preset dilution factors of the on-line samples and the limited measurability of excipients when using UV absorption for detection. Thakur et al. demonstrated the use of Near Infrared Spectroscopy (NIR) for monitoring and controlling protein and excipient concentrations during CFF in a conventional [14] and a single-pass setup [15]. Both applications are interesting as NIR is well suited for in-line applications in the manufacturing area [16]. However, the water absorbance is strong in the NIR [17] and Infrared Spectroscopy (IR) spectral region and shows a significant temperature dependence [11]. The chemometric model thus needs to be validated against temperature variations during a given process but also against long-term variations (e.g., seasonal fluctuations) [18]. Wasalathanthri et al. [19] used Fourier Transform Infrared Spectroscopy (FTIR) to monitor the protein and excipient concentration. While FTIR is more selective compared to NIR, the measurement time was 45s compared to the 15s presented by Thakur et al. with NIR. Both measurement speeds can be too slow for UF/DF runs if rapid concentration changes occur in processes due to large membrane areas.

In this study, Raman measurements were used to monitor the protein concentration and buffer exchange. Raman features advantages such as little interference from water and sharp spectral features for the different molecules. The results of the Raman measurements were compared to UV absorption and density measurements as a benchmark. As the changes in buffer and excipient concentrations during the DF are decreasing with increasing process time, an EKF was implemented to estimate the process state based on a semi-mechanistic process model with the predictions on Raman and density measurements. This setup was applied in three case studies to evaluate its performance in different processes and to show the benefits and the limitations of the setup.

## Materials and methods

### UF/DF experiments

#### Experimental setup

**Fig. 1:**
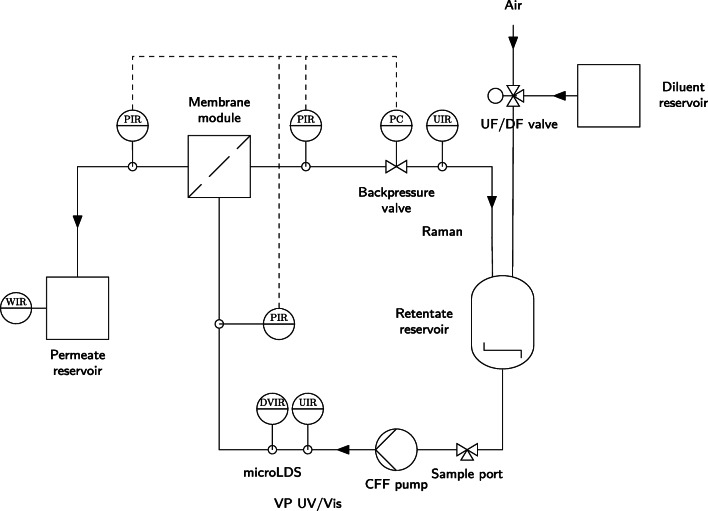
Piping and instrumentation diagram of the experimental setup. A VP UV/Vis spectrometer, a microLDS and a Raman probe are incorporated into the flow of the Tangential Flow Filtration (TFF). Additionally, a three-way valve is incorporated to change between UF and DF phase. All sensors are connected to a computer for capturing the data centrally. Electronic communication lines are indicated by dashed lines. The letters indicate: C control, D density, I indicate, P pressure, R record, U multivariable, V viscosity, W weight

The custom-made setup from Rüdt et al. [20] and Rolinger et al. [12] was adjusted for automation of the UF/DF process. Figure 1 shows the setup as a Piping and Instrumentation Diagram (P&ID). A KrosFlo KRIIi CFF unit (Spectrum Labs, Rancho Dominguez, USA) was equipped with a FlowVPE Variable Pathlength (VP) Ultraviolet and Visible (UV/Vis) spectrometer (C Technologies, Bridgewater, USA), a non-bypass version of a flow-through micro Liquid Density Sensor (microLDS) (TrueDyne Sensors AG, Reinach, CH), a MarqMetrix BioReactor Ballprobe (MarqMetrix, Seattle, USA) inserted into an in-house made flow cell for Raman measurements and a T-piece with injection plug (Fresenius Kabi, Bad Homburg, DE) placed after the retentate reservoir of the CFF unit for drawing samples for off-line analytics. The ball probe was connected to a HyperFlux PRO Plus 785 Raman analyzer with Spectralsoft 2.8.0 (Tornado Spectral Systems, Toronto, CA). Additionally, a fractionation valve of an Äkta prime (Cytiva, Chicago, USA) was connected to a relay module, which was controlled via a NI USB-6008 data acquisition device (National Instruments, Austin, USA) to switch between air and DF buffer. A Topolino magnetic stirrer (IKA Werke GmbH & Co. KG, Staufen im Breisgau, DE) and a stir bar ensured homogeneous mixing in the retentate reservoir.

#### Lysozyme

The protocol for the UF/DF process for Lysozyme (Hampton Research, Aliso Viejo, USA) from our previous publication [21] was slightly adjusted by changing the DF buffer to 50mM phosphate buffer (VWR Chemicals, Leuven, B) at pH 7.1. In short, the process consisted of an UF phase concentrating the protein from 10gL^− 1^ to 20gL^− 1^, a DF phase, where a buffer exchange from citrate buffer at pH 6.0 to a phosphate buffer at pH 7.1 occurred, and a second DF phase to achieve a final concentration of 40gL^− 1^.

#### mAb

The mAb UF/DF process was adjusted from our previous publication [21]. In the first UF phase, the filtered mAb stock solution at a concentration of 2.79gL^− 1^ was concentrated to 25gL^− 1^. A Pellicon 3 Cassette with an Ultracel membrane (type C screen with 3kDa cutoff, 88cm^2^ membrane area) in a Pellicon Mini Cassette Holder was used (both Merck) in the UF/DF setup. The process was run at a Transmembrane Pressure (TMP) of 1.5bar and a feed flow of 45mLmin^− 1^. In the DF phase, the solution was diafiltrated with eight Diafiltration Volumes (DVs) of DF buffer (250mM glycine, 25mM histidine at pH 5.8). In the second UF phase, the solution was concentrated to approximately 100gL^− 1^. The mAb was provided by an industrial partner who may not be disclosed due to the established confidentiality agreement.

#### bsAb

For the Antibody (bsAb), the membrane, TMP and feed flowrate settings from the mAb process were used. The bsAb stock solution (concentration 11.49gL^− 1^) was adjusted with a 2M Tris(hydroxymethyl)amino methane (TRIS) buffer to pH 7.1 and filtered before use. In a first UF step, the concentration was raised to 25gL^− 1^. Next, the solution was diafiltrated with eight DVs of DF buffer (2.2mM sodium phosphate, 1.3mM TRIS). A second UF step concentrated the product to approximately 80gL^− 1^. The bsAb was provided by an industrial partner who may not be disclosed due to the established confidentiality agreement.

### Data acquisition and analysis

During experiments, all integrated sensors and devices communicated with and were controlled (except for the Raman analyzer) by a custom-made application developed in MATLAB (version R2020a, The Mathworks, Natick, USA) and adapted from Rüdt et al. [20] and Rolinger et al. [12]. Besides connecting the devices and starting and stopping measurements, the application gathered the signals from the integrated sensors and calculated quality attributes and process parameters. Communication and control were performed through software libraries provided by the different instrument manufacturers. In contrast to the previous publications, no Graphical User Interface (GUI) was used to display the signals to save computational power. Data acquisition and analysis of the density and viscosity measurements, Raman measurements, and UV measurements were performed as described below.

#### UV absorbance measurements and processing

UV slope spectra were recorded from 280nm to 300nm for lysozyme, mAb, and bsAb with a resolution of 5nm. For concentration calculations, the absorbance at 280nm was used without scatter correction. The settings resulted in a measurement speed of 0.9min per spectrum. To improve the measurement speed, measuring at a wavelength of 280nm would be sufficient. Measuring more wavelengths can give information about the formation of aggregates in the solution, as large aggregate scatter increases the background scatter signal in the UV range.

#### Temperature and protein concentration correction of density measurements

In general, the density *ρ* of solutions is affected by the buffer components, protein concentration, and temperature. For the obtained data, this was important as the used microLDS dissipates a noticeable amount of heat into the measured liquid. To obtain comparable results, the measured viscosity and density were corrected to a standard process temperature yielding $\eta _{\mathrm {T_{0}}}$ and $\rho _{\mathrm {T_{0}}}$, respectively. As the temperature differences were relatively small (Δ*T* ≤ 5*K*), it was assumed that the deviations from the ideal solution behavior were neglectable [22–24]. The temperature correction was thus performed by cross-multiplication for viscosity and density measurements.

1 $$ \rho_{T_{0}} = \frac{\rho_{\text{water}, T_{0}}}{\rho_{\text{water},T}}\rho $$

This approach is similar to the temperature correction of the sedimentation coefficient performed in analytical ultracentrifugation [25, 26]. Reference values for the density/viscosity of water were obtained from the National Institute of Standards and Technology (NIST) chemistry webbook [27].

To calculate the buffer density $\rho _{buffer,T_{0}}$, the influence of the protein concentration on the density was subtracted from the temperature corrected density $\rho _{T_{0}}$.

2 $$ \rho_{buffer,T_{0}} = \rho_{T_{0}}- a_{prot} \cdot c_{prot} $$

where *c*_*prot*_ is the protein concentration and *a*_*prot*_ is a buffer-dependent factor, also referred to as partial specific volume of the protein. To obtain *a*_*prot*_ serial dilutions of the protein in buffer solutions were performed and *a*_*prot*_ was estimated as the slope of an ordinary linear regression of $\rho _{T_{0}} = \rho _{buffer,T_{0}} + a_{prot} \cdot c_{prot}$ since a linear relationship is expected [28]. As the applied buffer conditions in this paper are fairly narrow in terms of pH range and ionic buffer strength, only small changes in *a*_*prot*_ are expected during the DF phase [29]. We therefore used *a*_*prot*_ for the DF buffer as an approximation for the whole process phase.

#### Raman measurements

The laser power during acquisition was set to 495mW with an exposure time of 800ms and 10 acquisitions per spectrum for lysozyme and the bsAb. Due to the lower concentration of the mAb, an initial exposure time of 1200ms was chosen. As the mAb showed a significant level of background scattering, which increased with increasing mAb concentration, the exposure time was step-wise lowered, every time the maximum intensity reached the saturation limit of the detector. X-axis, Y-axis, and laser calibration were done before the experiment according to the manual.

For Partial-least Squares (PLS) modeling, Solo 8.9 (Eigenvector Research, Inc., Wenatchee, USA) was used. First, different spectral preprocessing steps were evaluated to improve the model prediction and linearity based on the recorded dilution series. However, the raw spectra provided the best model accuracy during cross-validation and initial optimization. Consequently, no spectral preprocessing was done and no wavelength selection was done. Only mean centering was applied as it is a standard treatment for spectral data. More information on the PLS models is provided in the Electronic Supplementary Material. For visualization purposes, the automatic asymmetric Whittaker Filter was used along with the Savitzky-Golay filter (15 points, second-order, no derivative) to remove the background/baseline signal and to smooth the data.

#### Extended Kalman filter implementation

An EKF was used to smooth the data during DF. The EKF concept was selected, because it is the classical concept for extending the Kalman filter concept to non-linear state transitions and observer models, where the direct derivation of the Hessian and Jacobian matrix is possible [30, 31]. However, other alternatives like Particle filters, the Unscented Kalman Filter, or an EKF based on a second-order Taylor expansion [32] would have been also a valid choice for smoothing the data during the DF phase. The basic idea behind the EKF is to combine measurements with a non-linear process model to estimate the current true state of the process. This approach also makes predictions into the future possible by leveraging the predictive abilities of the non-linear process model. Predictions may be used to timely terminate reactions, anticipate unwanted behavior or control the process in other ways.

For DF processes, the process may be approximated by the buffer exchange in a Continuously Stirred Tank Reactor (CSTR) under the assumption that the retentate flow is much bigger than the permeate flow and the process volume remains constant. We thus describe the buffer exchange in our CFF setup by following differential equation:

3 $$ \begin{array}{@{}rcl@{}} \frac{dc}{dt}=c_{in} \frac{F}{V}- c \frac{F\kappa}{V}, \end{array} $$

where *c* and *c*_*in*_ are the concentration of the considered species in the retentate tank resp. the DF buffer, *F* is the constant permeate flowrate, *V* is the constant volume of the retentate tank and *κ* is an empirical sieving coefficient. For free membrane passing ions, *κ* is close to 1 [33]. If a Donnan effect occurs, the sieving coefficient *κ* can increase or decrease depending on the kind of interaction between the ions of the excipient, protein and membrane [33]. For the differential equation integration and for the EKF transfer function, we assume that *κ* is constant over time. Since *κ* is recursively estimated by the EKF, the estimate may change over the course of the run. By integration from *t*_*k*− 1_ to *t*_*k*_, we obtain:

4 $$ \begin{array}{@{}rcl@{}} \frac{c_{in}}{\kappa} - c_{k}= \left( \frac{c_{in}}{\kappa} - c_{k-1}\right) \exp{\left( -\frac{\kappa F}{V}{\Delta} t\right)} \end{array} $$

with *c*_*k*− 1_ and *c*_*k*_ being the concentration at *t*_*k*− 1_ and *t*_*k*_, respectively, and Δ*t* being the step in time. Consequently, a buffer signal during DF follows an exponential decay towards a new steady-state concentration. It is worth noting that Eq. 4 can directly be used for the EKF as long as a measurement calibration is available. This allows to directly estimate the empirical sieving coefficient *κ* by the EKF. For the current application, the goal was to implement an EKF which does not require prior calibration. To this end, we now replace the concentrations *c* with the more general concept of a signal linearly correlated to the concentration. The signal may either be a Raman band intensity, a density measurement, or indeed also a buffer component concentration. The signal may either be increasing or decreasing depending on the nature of the measurement. Transforming Eq. 4 and lumping the signal terms $\frac {x_{in}}{\kappa } - x(t)={\Delta } x(t)$ results in:

5 $$ {\Delta} x(t_{k})= {\Delta} x(t_{k-1}) \exp{\left( -\frac{\kappa F}{V} {\Delta} t\right)}. $$

Starting from Eq. 5, the EKF is now implemented as described in [30]. Equation 6 is used to predict the state vector $\boldsymbol {\hat {x}}_{k | k-1}$ at the time point *k* based on the measurements up to the time point *k* − 1. The first entry in the state vector $\boldsymbol {\hat {x}}_{k | k-1}$ is the estimated delta buffer signal $\hat {x}_{1}=E({\Delta } x)$. $\hat {x}_{2}$ is the estimated buffer exchange rate $E\left (-\frac {\kappa F}{V} {\Delta } t\right )$. As discussed above, the model assumes that the buffer exchange rate $\hat {x}_{2}$ is constant over time. $\hat {x}_{3}$ is the estimated offset, i.e., the terminal signal height $E\left (\frac {x_{in}}{\kappa }\right )$. The offset of the measurement signal $\hat {x}_{3,k | k-1}$ and the buffer signal $\hat {x}_{1,k | k-1}$ is then used to predict the observation $\hat {z}_{k | k-1}$ with Eq. 7.

6 $$ \begin{array}{@{}rcl@{}} &\text{Predict state of buffer signal} & \boldsymbol{\hat{x}}_{k | k-1}= \left[\begin{array}{c} \hat{x}_{1,k -1 | k-1} \cdot e^{\hat{x}_{2,k -1 | k-1}} \\ \hat{x}_{2,k -1 | k-1} \\ \hat{x}_{3,k-1 | k-1} \end{array}\right] \end{array} $$

7 $$ \begin{array}{@{}rcl@{}} &\text{Predict state of observation} & \hat{z}_{k | k-1}= ~\hat{x}_{1,k | k-1} + \hat{x}_{3,k | k-1} \end{array} $$

Equation 8 is used to predict the covariance matrix ***P***_*k*|*k*− 1_ from the previous covariance matrix ***P***_*k*− 1|*k*− 1_ and the Jacobian matrix ***F***_*k*_ to linearize the state function on the local point by a first-order Taylor series expansion. The process covariance matrix ***Q***_*k*_ is added to account for the model uncertainty. *σ*_*v*_ is the covariance coefficient of the process error.

8 $$ \text{Predict covariance matrix} \boldsymbol{P}_{k | k-1}= \boldsymbol{F}_{k} \boldsymbol{P}_{k-1 | k-1} \boldsymbol{F}_{k}^{\intercal} + \boldsymbol{Q}_{k}  $$

$$ \begin{array}{@{}rcl@{}} \text{with} ~ \boldsymbol{F}_{k} = \left[\begin{array}{ccc} e^{-\hat{x}_{2,k -1 | k-1} } & -e^{-\hat{x}_{2,k -1 | k-1}} \cdot \hat{x}_{1,k -1 | k-1} & 0 \\ 0 & 1 & 0\\ 0 & 0 & 1 \end{array}\right] \end{array} $$

$$ \begin{array}{@{}rcl@{}} ~ \text{and}~ \boldsymbol{Q}_{k} = \left[\begin{array}{ccc} {\sigma_{v}^{2}} & 0 & 0 \\ 0 & 0 & 0\\ 0 & 0 & 0 \end{array}\right] \end{array} $$

The innovation covariance matrix ***S***_*k*_ is calculated via Eq. 9 based on the Jacobian of the sensor transfer functions [***H***_*k*_], the covariance matrix ***P***_*k*|*k*− 1_ and the sensor covariance matrix [***R***_*k*_]. *σ*_*w*_ is the covariance coefficient of the sensor error.

9 $$ \text{Predict innovation covariance} \boldsymbol{S}_{k}= \boldsymbol{H}_{k} \boldsymbol{P}_{k | k-1} \boldsymbol{H}_{k}^{\intercal} + \boldsymbol{R}_{k}  $$

$$ \begin{array}{@{}rcl@{}} \text{with} ~ \boldsymbol{H}_{k} = \begin{array}{c} 1 \\ 0 \\ 1 \end{array} \text{and} ~ \boldsymbol{R_{k}} = \begin{array}{ccc} {\sigma_{w}^{2}} & 0 & 0 \\ 0 & 0 & 0\\ 0 & 0 & 0 \end{array} \end{array} $$

Now, the Kalman gain ***K***_***k***_ can be calculated via Eq. 10 from the covariance matrix *P*_*k*|*k*− 1_ and the sensor transfer functions ***H***_***k***_, scaled by the innovation covariance matrix ***S***_***k***_.

10 $$ \text{Predict Kalman gain} \boldsymbol{K}_{k}= \boldsymbol{P}_{k} \boldsymbol{H}_{k}^{\intercal} \boldsymbol{S}_{k}^{-1}  $$

With the calculated Kalman gain ***K***_*k*_, the prediction of the state estimate $\boldsymbol {\hat {x}}_{k | k}$ and the covariance matrix ***P***_*k*|*k*_ can be updated via Eqs. 11 and 12, respectively.

11 $$ \begin{array}{@{}rcl@{}} \text{Updated state estimate} \quad \boldsymbol{\hat{x}}_{k | k}&=& \boldsymbol{\hat{x}}_{k | k-1} + \boldsymbol{K}_{k} (z_{k} - \hat{z}_{k | k-1}) \end{array} $$

12 $$ \begin{array}{@{}rcl@{}} \text{Updated covariance estimate} \quad \boldsymbol{P}_{k | k}&=& (I-\boldsymbol{K}_{k} \boldsymbol{H}_{k}) \boldsymbol{P}_{k | k-1} \end{array} $$

In principle, the peak height of the buffer component in question in the Raman spectrum may be used as an input signal for the EKF. To improve the prediction and reduce noise levels, Raman spectra were factorized by a Principal Component Analysis (PCA) and the principal component score of the buffer component was used as input for the EKF.

### Off-line analytics by SE-HPLC

The off-line Size Exclusion High Performance Liquid Chromatography (SE-HPLC) analytic was done according to our previous publication [21], with the difference that already mAb and bsAb samples with concentrations higher than 30gL^− 1^ were diluted 10-fold. bsAb samples were analyzed according to the protocol for the mAb.

## Results and discussion

In this study, three different case studies are investigated to compare Raman spectroscopy, UV spectroscopy, and density measurement for their ability to measure the protein concentration and buffer exchange progress. First, the Raman spectra are discussed in detail. Then, Raman spectroscopy and UV spectroscopy are compared towards their prediction accuracy for the protein concentration. Finally, density measurements and Raman spectroscopy will be compared towards their ability to monitor the buffer exchange progress.

### Raman spectra

**Fig. 2:**
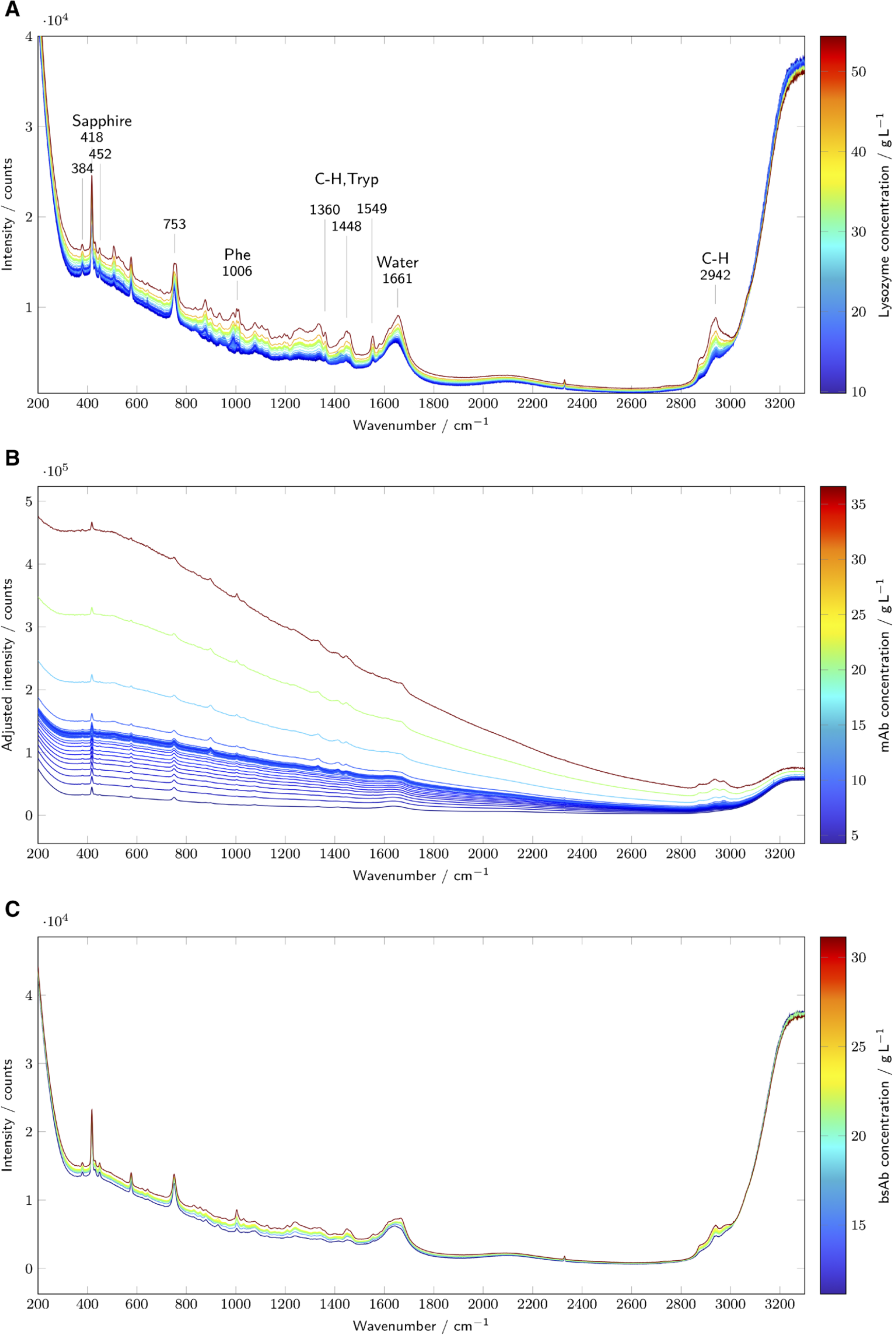
The raw Raman spectra recorded by the in-line Raman analyzer are plotted and colored according to the protein concentration. The different subplots show the results for lysozyme (A), mAb (B), and bsAb (C)

In Fig. 2, every 50th spectrum of the lysozyme process and every 40th spectrum of the mAb and bsAb process are shown. For lysozyme, the protein features are well visible in the Raman spectra with bands in the range from 500cm^− 1^ to 1700cm^− 1^ and around 2900cm^− 1^. The sapphire bands at 384cm^− 1^, 418cm^− 1^, 452cm^− 1^ and 753cm^− 1^ are visible in the Raman spectra of all case studies and are, as expected, constant. The protein bands, especially at 1006cm^− 1^ originating from phenylalanine, 1360cm^− 1^, 1448cm^− 1^, and 1549cm^− 1^ originating from tryptophane and C-H deformation [34, 35], and at 2942cm^− 1^ originating from C-H stretching [25], are distinct from other components by the fact that they also increase during the second UF step. Additionally, an increase in background signal correlates with the increase in protein concentration. This phenomenon was already discussed in a previous publication and is likely related to increased Rayleigh scattering [36]. For the bsAb, the spectra look comparable to the lysozyme spectra, even though the height of the protein features is lower. The mAb shows an increased background signal in comparison to the other two proteins and, therefore, lower intensities in the protein bands compared to the background signal. Already in the last publication, an increased molecule to molecule interaction of the mAb was detected, which lead to buffer-induced light scattering increase and gel formation [36]. Here, the change in the background signal is more pronounced than the change in protein features. Again, the intensity of the background signal correlates with the protein concentration. Measurement-wise, the large amount of background signal made a reduction of exposure time necessary to prevent the over-saturation of the detector. The spectra were subsequently normalized by the exposure time (shown in Electronic Supplementary Material).

### In-line protein concentration measurements

**Fig. 3:**
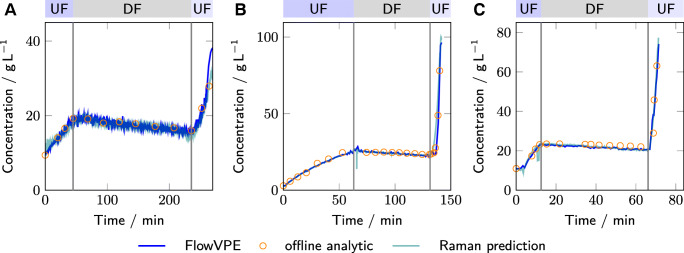
The total protein concentration is shown as measured by the in-line FlowVPE VP spectrometer (blue lines), Raman analyzer (teal lines) and off-line SE-HPLC (orange circles). The different subplots show the results for lysozyme (A), mAb (B), and bsAb (C)

For monitoring the protein concentration, the absorbance at 280nm from the VP UV spectrometer and the full spectra of the Raman analyzer in combination with a PLS model were used. In Fig. 3, the predicted protein concentrations are compared to the results obtained from off-line SE-HPLC analysis. Qualitatively, both the predicted protein concentration from the Raman analyzer and the VP UV spectrometer are in good agreement with the off-line analytics for all three processes. For lysozyme, towards the end of the second UF, the FlowVPE signal starts to deviate from the Raman signal. This was attributed to an increasing amount of air bubbles in the solution, which impaired the FlowVPE measurements.

During the whole process, the Raman predictions showed a few outliers, probably caused by air bubbles in the measurement chamber. In manufacturing, this could be mitigated by rejecting the predictions based on the Hotelling’s *T*^2^ value or the distance to the model hyperplane. The PLS model for the Raman-based protein concentration predictions is mostly influenced by the background signal. This is in agreement with [36] and can already be seen by comparing the concentration prediction to Fig. S1 in the electronic supplementary material, which shows the intensity trend at 700cm^− 1^, where no protein vibrational band is located. Theoretically, a PLS model might not be necessary to predict the protein concentration as a single intensity already correlates well to the protein concentration. However, a single wavenumber/wavelength measurement has usually lower accuracy compared to the PLS model based on several wavenumber [36]. The dependence of the PLS model on the background reduces the specificity of the model for the protein of interest. For example, an increased aggregate content likely increases the background signal disproportionately and thereby affects the protein concentration prediction. In routine production, the reduced selectivity is however not a problem since any manufacturing process must be reproducible regarding the feed composition and will work with highly pure protein solution, especially towards the end of the process. UV/Vis absorption relies on the more specific absorption of the aromatic amino acids [11]. Quantification is normally robust and not significantly impacted by batch-to-batch variability. In the current experimental results, the UV-based protein concentration measurements show fewer outliers in comparison to the Raman measurements. However, UV measurements were already filtered based on a coefficient of determination higher than 0.97 during the VP regression.

Quantitatively, the Root Mean Square Error (RMSE) for lysozyme and the bsAb of the UV- and Raman-based measurements are very similar (cf. Table 1). However, for the mAb, the Root Mean Square Error (RMSE) of the Raman predictions is with 4.59gL^− 1^ more than twice as high as the RMSE of the UV-based measurements at 1.73gL^− 1^. This difference is mostly driven due to the residuals in the second UF phase. Due to the fast change in protein concentration, the uncertainty in the sampling time affects the measurement accuracy more strongly than during the rest of the process. Furthermore, as shown in the P&ID (Fig. 1), the Raman spectra were measured in the retentate (due to pressure constraints of the flow cell), while sampling and UV-based measurements were conducted in the feed. Normally, if the feed and retentate flow are similar, this does not pose a problem. However, at the beginning of the second UF phase, the process progressed very quickly introducing a systematic offset and increasing the overall RMSE for the Raman measurements.

**Table 1:** RMSE and coefficients of determination for UV and Raman measurements compared to off-line analytics

	Concentration prediction based on FlowVPE			Concentration prediction based on Raman		
	RMSE/ g L^− 1^	*R*^2^	normalized RMSE %	RMSE/ g L^− 1^	*R*^2^	normalized RMSE %
Lysozyme	0.87	0.9874	4.87	0.99	0.9799	5.54
mAb	1.73	0.9943	3.31	4.59	0.9788	8.80
bsAb	2.88	0.9709	3.83	2.67	0.9771	3.55

Given the results obtained in the three case studies, both VP UV/Vis spectroscopy and Raman spectroscopy are useful tools for quantifying proteins in-line in real-time during UF/DF processes. Raman spectroscopy was quicker compared to VP UV/Vis spectroscopy, which takes about 8s when measuring at one wavelength at four pathlengths. UV/Vis spectroscopy may be more robust towards process variability (e.g., changing aggregate content), because the background effect in the Raman measurements seems to mostly origin from the molecular weight and interaction between molecules. Additionally, UV/Vis spectroscopy works by simple determination of the absorption coefficient without the need to calibrate a chemometric model. Although the data analysis of the Raman spectra is more complex in comparison to UV/Vis measurements, Raman spectroscopy allows for simultaneous insights into the protein and excipient concentrations. The ability of Raman spectroscopy to selectively measure different excipients will be used in Section 3 to monitor the buffer exchange process. Both methods measure protein concentration in the investigated range without any major deviations from linearity. Noise levels remain comparably small. In this study, the traditional limit of quantification could not be applied for comparing the concentration predictions. This is due to the fact that the concentration predictions from Raman spectra relied on a multivariate PLS model which does not permit traditional limit of quantification calculations. It is worth considering that the limit of quantification for Raman-based concentration prediction changes due to changing measurement settings (e.g., exposure time). We therefore consider the comparison of Root Mean Square Error of Cross-Validation (RMSECV) as most insightful.

Predicting the concentration of the different aggregate and fragment species was attempted with Raman spectroscopy in this study, but it was ultimately unsuccessful. The concentrations of the individual species might be too low and the structural changes between differently sized species not prominent enough to be picked up by Raman spectroscopy in the short measurement times. However, Wei et al. [37] showed promising results to quantify aggregates and fragments with a multi-product PLS model based on offline Raman measurements with a measurement time of 22.5 minutes.

### Buffer exchange progress monitoring

**Fig. 4:**
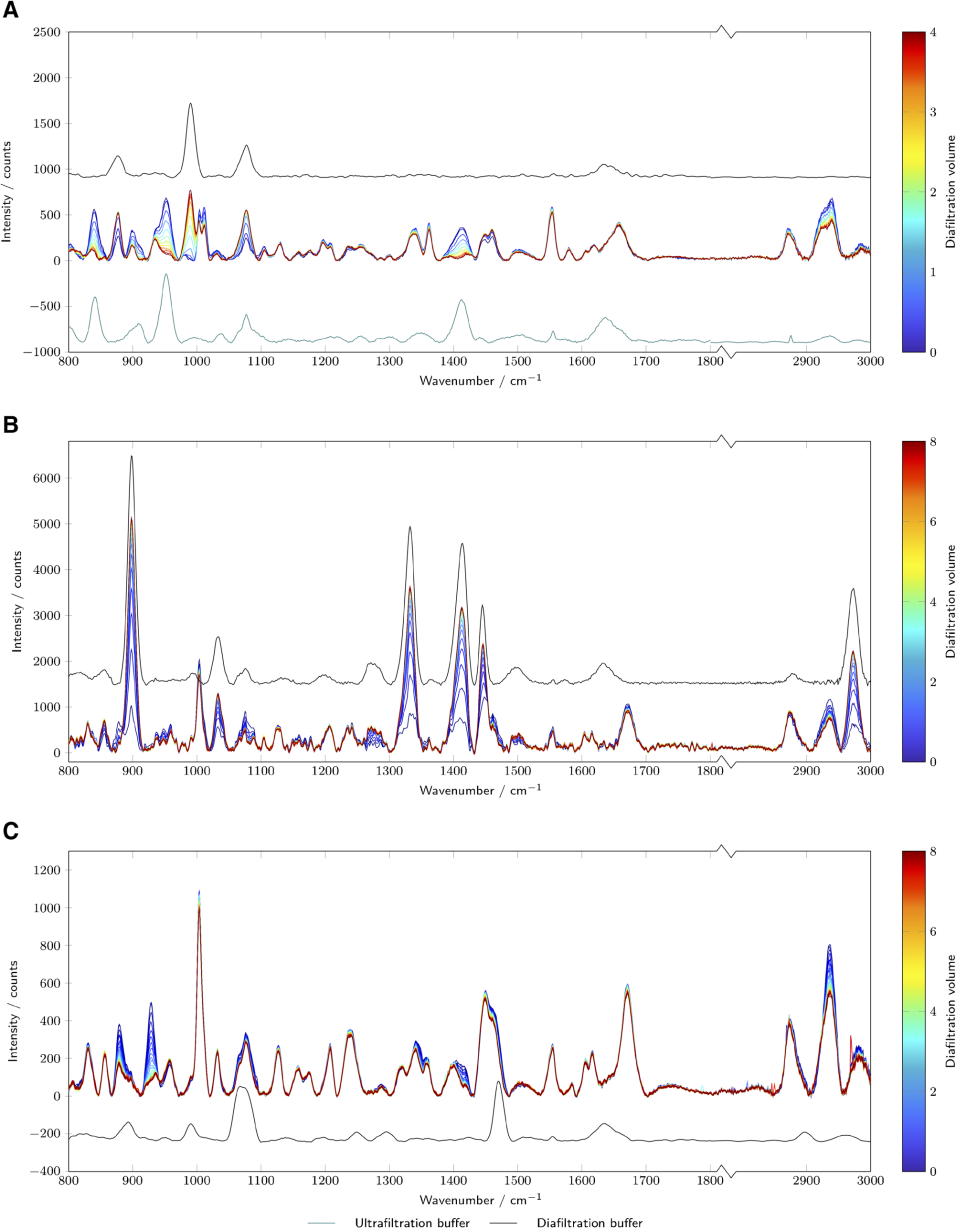
The preprocessed Raman spectra recorded during the DF phase are plotted and colored by diafiltration volumes. The diafiltration buffer (black line) are plotted with an offset. For lysozyme, additionally the ultrafiltration buffer (teal line) is depicted. The different subplots show the results for lysozyme (A), mAb (B), and bsAb (C)

In Fig. 4, the preprocessed Raman spectra of the DF phase are plotted. For the lysozyme case study, the change from citrate buffer to phosphate is most prominently visible at 840cm^− 1^, 952cm^− 1^, 990cm^− 1^ and 1412cm^− 1^. The citrate buffer has a significant number of bands (see teal line). The most prominent band at 952cm^− 1^ can be attributed to COOH out-of-plane deformation vibration of the carboxylic acid group [38]. Also prominent is the carboxylate symmetric stretching band at 1412cm^− 1^ and the carbon-carbon stretching mode at 840cm^− 1^ [39]. Phosphate shows a major band at 990cm^− 1^ due to the P-O stretching of phosphate [38, 40–42] along with bands at 1078cm^− 1^ and 877cm^− 1^, which can be attributed to the symmetrical P(OH)_2_ stretching vibration and the in-plane PO_2_ [41].

For the mAb case study, the DF buffer consists of histidine and glycine which have the most dominant peaks at 899cm^− 1^, 1332cm^− 1^, 1413cm^− 1^, 1446cm^− 1^ and 2972cm^− 1^ (Fig. 4B, black line). Glycine has a strong C-C stretching band at 899cm^− 1^ [43]. The other two intense Raman bands 1332cm^− 1^ and 1413cm^− 1^ can be attributed to the twisting of the NH_3_ and CH_2_ groups and a NH_3_ wagging mode coupled with COO stretching [43]. A smaller band is located at 1448cm^− 1^ and is caused by CH_2_ scissoring [43]. The peak at 2972cm^− 1^ is caused by the symmetric stretching of CH_2_ [44]. These peaks are expected to build up during the DF. No distinct bands for histidine are visible. This might be caused by the 10-fold lower concentration.

**Fig. 5:**
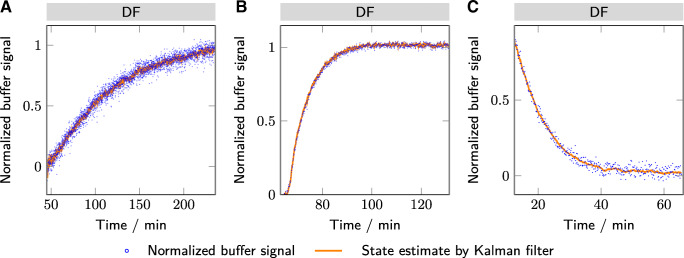
The normalized buffer signal derived from a PCA of the Raman spectra are plotted over time with the state estimate of the Kalman filter. The different subplots show the results for lysozyme (A), mAb (B), and bsAb (C)

The phosphate and TRIS DF buffer for the bsAb case study is very low concentrated, therefore no changing peaks attributed to the DF buffer are visible in the process spectra. TRIS has a C*H*_2_ deformation band at 1470cm^− 1^ and CO stretching at 1066cm^− 1^ [38, 45]. However, a chemical with two peaks at 881cm^− 1^ and 930cm^− 1^ is depleted during the DF. This chemical presumably originates from the previous production step, a chromatographic separation.

Figure 5 shows the normalized buffer signal derived from the Raman spectra over time. The normalized signal consists of the normalized scores of the principal component collecting the spectral variability due to the buffer exchange. This approach was used to improve the signal-to-noise ratio. In principle, also a unique peak of a buffer component could be chosen. However, due to the various proteineous Raman peaks, the peaks are often overlapping with the protein peaks. A PCA allows separating the protein signal from the buffer signal. The PCA score plots are shown in the Electronic Supplementary Material.

For the first case study with lysozyme, the phosphate peaks of the DF buffer are too weak and overlapping with the citrate peak, so that an individual monitoring of the two species is not possible. Instead, the principal component representing the citrate buffer was used. The signal itself follows a decay curve as expected during DF. Interestingly, the signal is still changing around the end of the DF at four DV. Raman spectroscopy provides this information in real-time allowing for an immediate evaluation of the DF process. Based on the observed behavior, a decision may be taken to extend the DF phase.

For both the mAb and bsAb, the buffer signal seems stable towards the end of the DF. For the mAb, the principal component analysis did not differentiate between the two components of the DF buffer, glycine and histidine. Depending on the net-charge of the mAb at the given pH, a Donnan effect was previously observed with an accumulation of histidine during the DF for negatively charged (mAbs) [46, 47]. The observed accumulation was within 3mM after eight DV [46]. Either the higher overall ion-concentration [48], a positively charged mAb or the quantification limit of the Raman could have led to the non-observability of the effect.

**Fig. 6:**
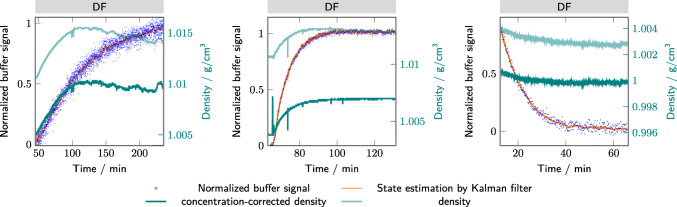
The density (light teal) and the concentration-corrected density (teal) are plotted over the DF run time along with the normalized buffer signal from the Raman measurements and the EKF prediction (orange). The different subplots show the results for lysozyme (A), mAb (B), and bsAb (C)

The Raman signal in all case studies shows significant noise, which makes the signal more difficult to interpret and to use to control the process. To reduce the noise level, an EKF was used to approximate the real process state from the noisy measurements. Kalman filters allow for some plant variability. They are also applicable in real-time for recursive state estimation and control. The orange lines in Figs. 5 and 6 indicate the EKF-filtered results. It is worth noting that the EKF successfully suppresses a significant part of the measurement noise. Furthermore, with the used estimates for the system and measurement noise, the EKF is still flexible enough to adjust the prediction dynamically to changing conditions. For example, during the diafiltration of the mAb (Figs. 5B and 6B), the buffer exchange initially starts more slowly than expected. The EKF incorporates this into its prediction by adjusting the two other state variables (offset and exponential decay constant). The estimated state variables may also be used to gain insight into changes of the filtration behavior, e.g., due to a changing sieving coefficient. This approach provides a real-time mechanistic insight into the process performance and may help to improve the understanding of the ongoing process. The EKF, thus, provides an interesting tool for real-time recursive evaluation of the buffer exchange progress and a method for an improved understanding of the ongoing process variability.

**Fig. 7:**
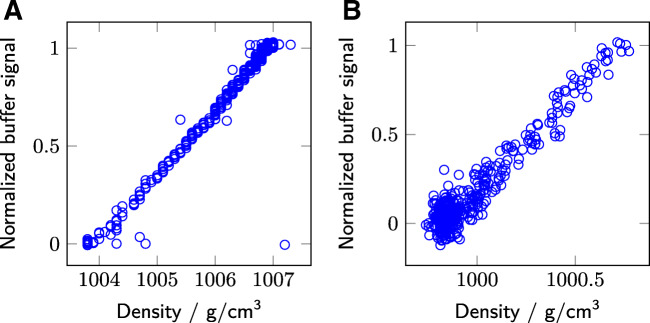
The concentration- corrected density is compared to the normalized Raman signal. The different subplots show the results for mAb (A), and bsAb (B)

Next to the Raman signal, the buffer exchange was monitored by the evolution of the density signal. The density signal is univariate, collecting information from all components in the solution in one variable. Therefore, no separate monitoring of individual species is possible. Next to the buffer components, the density signal is also affected by changing protein concentrations. In Fig. 6, the temperature and protein concentration-corrected density are plotted. The concentration correction was done with the protein concentration predictions from the Raman due to the higher measurement frequency of the Raman signal. For lysozyme, the density measurements were corrupted by air bubbles due to the increasing viscosity of the solution over time. Repeating the experiments led to the same phenomena. The air bubbles seem to decrease the liquid density and remain in the feed due to the viscosity of the solution. Only filters could help to remove bigger air bubbles from the solution, but might introduce more aggregation due to shear forces. For viscose protein solutions, the density measurements seem difficult due to the air entrapment. Therefore, the concentration correction does not work for the lysozyme case study, because during the DF the corrected density is decreasing, which is not physically reasonable and not in agreement with the Raman data. For the mAb and bsAb case study, the protein concentration correction of the density leads to a stable signal towards the end of the DF. The measured density for the mAb and bsAb case study seems to decline over the whole DF phase due to the protein concentration decrease, whereas the Raman-based buffer signal already indicates a stabilization and thereby completion of the DF process. The measured density signal alone is, therefore, only of limited use to monitor and control the DF phase. The protein concentration correction density seems to agree with the Raman-based buffer signal. However, a direct comparison between the protein concentration-corrected density and the normalized buffer signal by Raman is difficult to make based on Fig. 6. Therefore, the comparison is directly plotted in Fig. 7.

Figure 7 shows the comparison between the Raman measurements and the concentration-corrected density measurements. The lysozyme data is not plotted due to the unreliability of the density measurements as discussed above. Both are in good agreement, even though a significant noise level is apparent for both measurements. For the density data, the Kalman filter can improve the DF progress prediction as well. The density signal has the benefit of even observing Raman-inactive components in the solution, like NaCl, under the prerequisite of a density difference between buffers. However, the needed protein concentration correction makes a second sensor necessary, which adds complexity and room for failure.

## Conclusion

In this study, the advantages and disadvantages of Raman spectroscopy for monitoring UF/DF processes were shown in three case studies and compared to UV absorption and density measurements as a benchmark. To improve the sensitivity of the measurements, an EKF was implemented to estimate the process state during the DF based on a semi-mechanistic process model combined with the predictions of Raman and density measurements. Raman spectroscopy and VP UV/Vis spectroscopy were compared for their prediction accuracy of the protein concentration in comparison to off-line measurements. VP UV spectroscopy showed slightly better or comparable coefficients of determination in comparison to the Raman measurements. UV concentration measurements were derived based on the absorption coefficient at 280nm, while Raman measurements required a PLS model to predict the protein concentration. Raman measurements took less than a second in comparison to eight seconds for the VP UV measurements. The higher measurement speed of the Raman spectrometer may be an advantage for fast processes. However, the Raman measurements were more prone to outliers in comparison to the UV measurements. A drawback of the Raman spectroscopy is that the prediction of the protein concentration seems to rely on the unspecific background effect, that correlates with the protein concentration. In addition to the protein concentration prediction, the Raman spectra provided the concentration of Raman-active buffer components. These concentration predictions were used to monitor the buffer exchange progress. To reduce the measurement noise, an EKF was used for state estimation. The prediction of the buffer exchange progress by Raman was less noisy compared to the density measurement. Another advantage of Raman spectroscopy is the ability to monitor individual buffer components.

Among other applications, Raman measurements thus pave a further step on the way towards the real-time control of the protein concentration during and at the end of the UF/DF process ensuring the final product concentration and buffer composition within the processes. Raman measurements thus pave a further step on the way towards Real-time Release Testing (RTRT) by replacing off-line in-process controls of critical quality attributes by their in-line equivalents.

### Electronic supplementary material

Below is the link to the electronic supplementary material.

(PDF 1.03 MB)
