# Correction to: Monitoring of ultra- and diafiltration processes by Kalman-filtered Raman measurements

**DOI:** 10.1007/s00216-024-05168-1

**Published:** 2024-02-22

**Authors:** Laura Rolinger, Jürgen Hubbuch, Matthias Rüdt

**Affiliations:** 1 Institute of Engineering in Life Sciences, Section IV: Biomolecular Separation Engineering, Karlsruhe Institute of Technology (KIT), Fritz-Haber-Weg 2, 76131 Karlsruhe, Germany; 2 Hoffmann-La Roche AG, Basel, Switzerland; 3 Institute of Life Technologies, HES-SO Valais-Wallis, Rue de l’industrie 19, Sion, 1950 Switzerland

**Correction to: Analytical and Bioanalytical Chemistry (2023) 415: 841–854**

**https://doi.org/10.1007/s00216-022-04477-7**

The authors regret that the sign convention in the description of the extended Kalman filter in the original publication was flawed. The state vectors $\hat{x}_1$ and $\hat{x}_2$ should have been defined as $\hat{x}_1=E(-\Delta x)$ and $\hat{x}_2=E(\frac{\kappa F}{V}\Delta t)$, respectively. Note that the sign of both state variables was changed. Consequently, a minus sign is missing in the exponential function of the transfer function for the first state estimate (Eq. 6 in the original publication), i.e.

1 $$\begin{aligned} \hat{\textbf{x}}_{k \vert k-1}= \begin{bmatrix} \hat{x}_{1,k -1 \vert k-1} \cdot e^{-\hat{x}_{2,k -1 \vert k-1}} \\ \hat{x}_{2,k -1 \vert k-1} \\ \hat{x}_{3,k-1 \vert k-1} \end{bmatrix} \end{aligned}$$

Two minor notation errors shall furthermore be disclosed: $\textbf{H}_k$ should be a row vector (defined as a column vector) and $R_k = \sigma ^2_w$ is a scalar. All other equations are correctly documented and not impacted by the disclosed errors.

As an immediate consequence of the errors coming to our awareness, we reviewed the code developed for this project. We conclude that the errors were limited to the mathematical description in the paper. The code is not affected and was correctly implemented from the beginning. Therefore, all visualizations and conclusions drawn in the paper remain valid.

For simplifying the understanding of the implemented extended Kalman filter for future readers, we are publishing a Python-based version of the code along this correction.

### Supplementary Information

Below is the link to the electronic supplementary material.

Supplementary file 1 (csv 60 KB)

Supplementary file 2 (ipynb 447 KB)

Supplementary file 3 (zip 94 KB)

