# Supplementary material for: Monitoring of ultra- and diafiltration processes by Kalman-filtered Raman measurements
Source: Anal Bioanal Chem. 2023 Jan 18;415(5):841–54. doi: 10.1007/s00216-022-04477-7 (PMC9883314; doi:10.1007/s00216-022-04477-7)
Supplement: Supplementary file 1 — (PDF 1.03 MB) [file 216_2022_4477_MOESM1_ESM.pdf]

Monitoring of Ultra- and Diafiltration Processes by Kalman-filtered Raman Measurements - Supplementary Material

Laura Rolinger, Jürgen Hubbuch, Matthias Rüdt

S1 Exposure time correction

In Figure S1, the Raman intensity at $700\,\mathrm{cm}^{-1}$ and the exposure time-adjusted Raman intensity at $700\,\mathrm{cm}^{-1}$ are plotted over time for the monoclonal Antibody (mAb) run. The exposure time adjustment yields a fairly smooth curve, which correlates to the protein concentration in the run, even though there is no protein band at $700\,\mathrm{cm}^{-1}$. It seems, that the increase in background signal is mainly driven by the protein concentration. It is interesting to note that the background effect seems not be influenced by the buffer change during the Diafiltration (DF) phase.

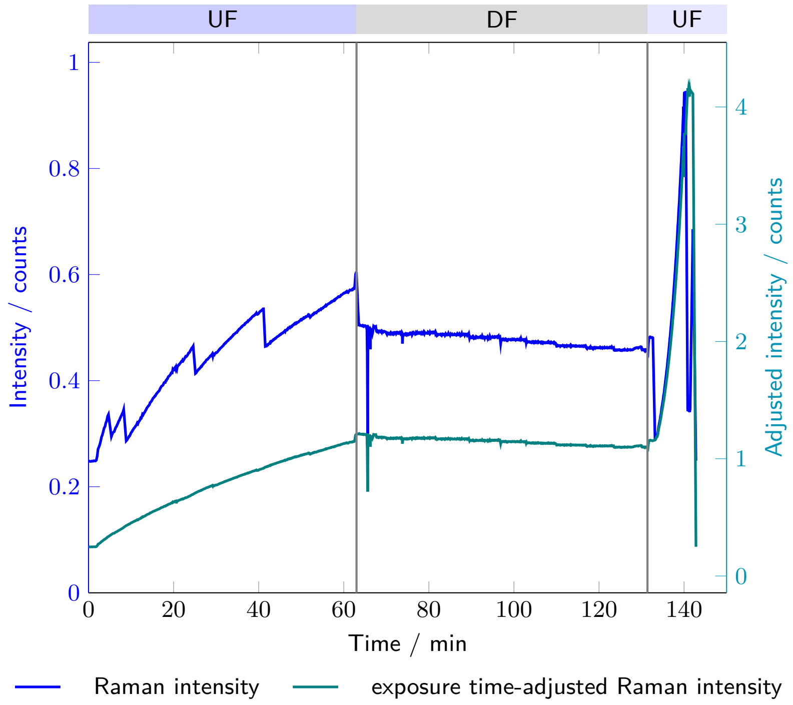

Figure S1: The Raman intensity at $700\,\mathrm{cm}^{-1}$ and the exposure time-adjusted Raman intensity at $700\,\mathrm{cm}^{-1}$ are plotted during the mAb run.

S2 Additional information on Raman-based PLS models

S2.1 Selection of number of Latent Variables

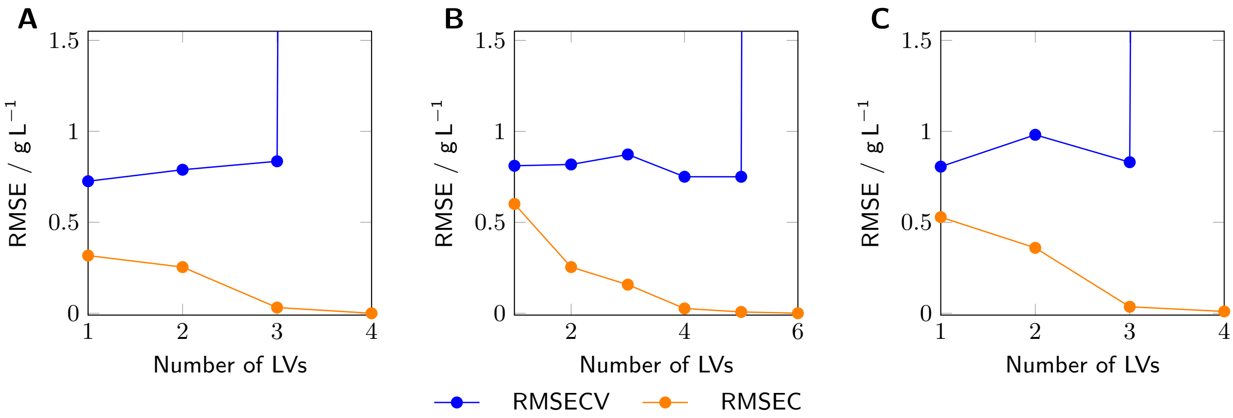

Figure S2: The RMSECV and RMSEC are plotted over the number of LVs for the Raman-based PLS models. The different subplots show the results for lysozyme (A), mAb (B), and bsAb (C).

In Figure S2, the Root Mean Square Error of Cross-Validation (RMSECV) and Root Mean Square Error of Calibration (RMSEC) over the number of Latent Variables (LVs) is shown. It can be seen, that increasing the number of LVs above one does not improve the prediction ability of the model. The main concentration information seems to be already captured in the first latent variable.

S2.2 Preprocessing evaluation

Table S1: Inlfuence of different preprocessing options on RMSEC and RMSECV of the Raman based PLS model for the mAb

Preprocessing	Number of LVs	Wavenumber range / cm^{-1}	RMSEC / $g\,L^{-1}$	RMSECV / $g\,L^{-1}$
Mean center	1	200-3300	0.60	0.81
1st deriv., mean center	1	200-3300	2.53	3.47
2st deriv., mean center	1	200-3300	5.27	11.52
EMSC, mean center	1	200-3300	9.33	12.59
Mean center	1	300-1800	0.60	0.80
1st deriv., mean center	1	300-1800	3.16	4.68
2st deriv., mean center	1	300-1800	5.18	9.18
EMSC, mean center	1	300-1800	11.43	15.45
1st deriv., mean center	2	300-1800	0.91	1.54
2st deriv., mean center	2	300-1800	1.44	7.78

As the Raman spectra for the mAb mainly contain the background information, which is correlated to the protein concentration, the model accuracy is reducing, when removing more of the background information. Either by removing the background through preprocessing or by reducing the wavelength range. When removing the background effect, an increase in LVs improves model prediction. More preprocessing options could be evaluated to further improve the model performance, for instance by using a Genetic Algorithm (GA). However, the Partial-least Squares (PLS) model calibration was based on a simple dilution series resulting in a small calibration data set. Therefore, a simple model was built to fulfill the prediction requirements.

Table S2: Inlfuence of different preprocessing options on RMSEC and RMSECV of the Raman based PLS model for the bsAb.

Preprocessing	Number of LVs	Wavenumber range / cm^{-1}	RMSEC / $g\,L^{-1}$	RMSECV / $g\,L^{-1}$
Mean center	1	200-3300	0.53	0.81
1st deriv., mean center	1	200-3300	0.41	0.80
2st deriv., mean center	1	200-3300	0.13	2.01
EMSC, mean center	1	200-3300	1.29	1.99
Mean center	1	300-1800	0.52	0.85
1st deriv., mean center	1	300-1800	0.38	0.82
2st deriv., mean center	1	300-1800	0.53	0.80
EMSC, mean center	1	300-1800	1.70	3.25

Table S3: Inlfuence of different preprocessing options on RMSEC and RMSECV of the Raman based PLS model for Lysozyme.

Preprocessing	Number of LVs	Wavenumber range / cm^{-1}	RMSEC / $g\,L^{-1}$	RMSECV / $g\,L^{-1}$
Mean center	1	200-3300	0.53	0.81
1st deriv., mean center	1	200-3300	1.27	2.53
2st deriv., mean center	1	200-3300	1.51	4.65
EMSC, mean center	1	200-3300	0.40	1.24
Mean center	1	300-1800	0.26	0.90
1st deriv., mean center	1	300-1800	1.53	2.71
2st deriv., mean center	1	300-1800	0.91	2.60
EMSC, mean center	1	300-1800	1.88	3.05

S2.3 Scores plot

In Figure S3, the scatter plots show the scores on the first Principal Component (PC) against the scores on the second and third PC. The first PC always contains mainly the concentration information. The second PC seems to be mostly influenced by the buffer exchange.

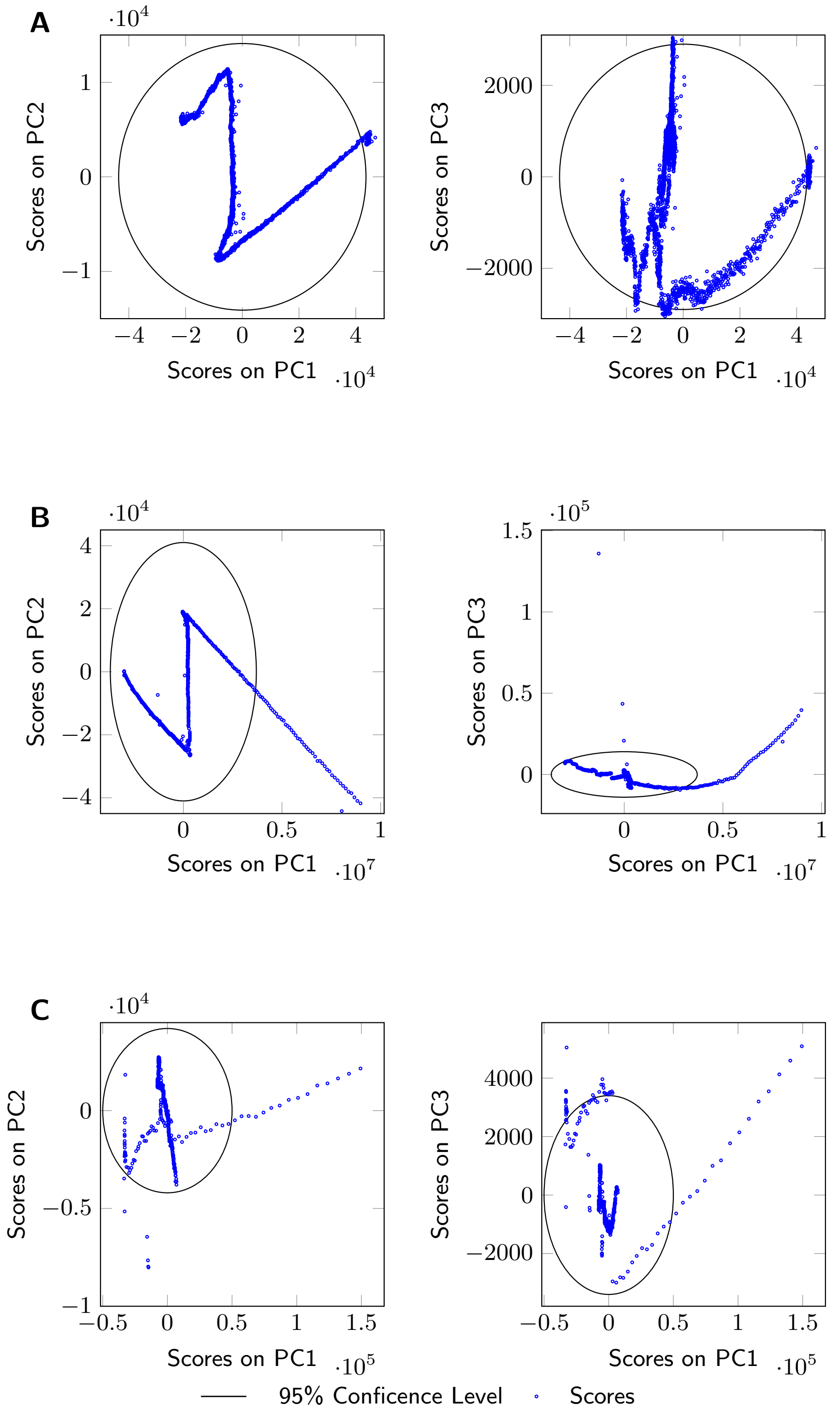

Figure S3: Scatter plots of scores from PCA. The different subplots show the results for lysozyme (A), mAb (B), and bsAb (C).
